# The role of media and community engagement in COVID-19 vaccinations in Tanzania

**DOI:** 10.4102/jphia.v16i3.705

**Published:** 2025-04-18

**Authors:** Ambrose T. Kessy, Chima E. Onuekwe, William M. Mwengee, Haonga Tumaini

**Affiliations:** 1 Directorate of Research, Publications and Consultancy, The University of Dodoma, Dodoma, United Republic of Tanzania; 2 Department of Planning, Finance and Administration, The Law School of Tanzania, Dar es Salaam, United Republic of Tanzania; 3 Department of Immunization, Emergency Preparedness and Response, World Health Organization, Dodoma, United Republic of Tanzania; 4 Centre for Health and Allied Legal and Demographical Development, Research and Training (CHALADDRAT), Nnamdi Azikiwe University, Awka, Nigeria; 5 Department of Immunization/Vaccination, Emergency Preparedness and Response, World Health Organization, Dar es Salaam, United Republic of Tanzania; 6 Department of Health Promotion, Ministry of Health, Dodoma, United Republic of Tanzania

**Keywords:** communication strategies, COVID-19 vaccine uptake, Tanzania, media, community outreach, vaccine acceptance, public perception, misinformation, vaccine hesitancy

## Abstract

**Background:**

The COVID-19 pandemic underscored the critical role of communication strategies in influencing public health behaviours, particularly vaccine uptake. In Tanzania, media and community engagement strategies have been pivotal in efforts to enhance COVID-19 vaccine acceptance; however, their effectiveness remains under scrutiny.

**Aim:**

This study examines the role of media and community engagement in promoting COVID-19 vaccine acceptance in Tanzania by analysing various communication channels and community outreach initiatives.

**Setting:**

The study was conducted in Tanzania across eight purposively selected regions that encompass a broad spectrum of socio-cultural contexts and infrastructural variations.

**Methods:**

A mixed-methods approach was employed, combining quantitative surveys (*N* = 3098), qualitative interviews and focus group discussions (*N* = 30) across eight regions. Stratified random sampling ensured proportional representation, while thematic analysis identified key trends in perceptions, vaccine uptake and the influence of the media and community leaders.

**Results:**

High levels of awareness (99.3%) regarding COVID-19 and vaccination were observed; however, vaccine uptake was uneven, with 37.2% vaccinated and regional variations persisting. Trusted sources included traditional media (radio and television) and community influencers, whereas misinformation and inconsistent government messaging contributed to hesitancy. Community engagement by healthcare providers and local leaders significantly influences vaccine acceptance.

**Conclusion:**

These findings highlight the importance of context-specific communication strategies that integrate media and community engagement to build trust, address misinformation and increase vaccine uptake. Policymakers and public health professionals should focus on fostering consistency in messaging, leveraging local leaders and tailoring outreach to diverse socio-cultural contexts.

**Contribution:**

This study makes a substantial contribution by empirically demonstrating the critical roles that media and community engagement play in shaping vaccine acceptance in Tanzania.

## Introduction

The COVID-19 pandemic, caused by the novel coronavirus severe acute respiratory syndrome coronavirus 2 (SARS-CoV-2), has presented unprecedented global health challenges since its emergence in late 2019. In response to the pandemic, the development and distribution of vaccines have been prioritised globally as a primary strategy to mitigate the impact of the virus. The success of vaccination campaigns relies heavily on public acceptance and uptake, which are influenced by various factors including media coverage and community engagement.^1^ However, there is noticeable hesitance regarding vaccine uptake among people, which raises concerns about whether media and community engagement have played key roles. Initial scepticism towards COVID-19 vaccines fuelled by misinformation and distrust in public health directives has been a critical barrier.^2,3^ This scepticism underscores the importance of effective communication strategies for increasing vaccine uptake. The media has played a crucial role in shaping public perception, with misinformation and conflicting information contributing to vaccine hesitancy.^4^ Transparent and effective communication, along with proactive community involvement, are key strategies for increasing vaccine acceptance and addressing the complex interplay between factors affecting hesitancy.^5^

While the global response to COVID-19 centred on vaccination as a key strategy, Tanzania faced unique challenges in vaccine uptake influenced significantly by media and community engagement.^6^ In Tanzania, as in many other countries, these factors played a key role in shaping public perceptions and behaviours towards COVID-19 vaccination. The COVID-19 vaccine hesitancy in Tanzania is shaped by multiple sociopolitical and vaccine-related factors. A study conducted in rural and urban Tanzania revealed that concerns over vaccine safety, limited knowledge about vaccines and fear of the vaccine’s impact on preexisting conditions were significant contributors to hesitancy.^7^ Political factors, including inconsistent messages from communities and political leaders, as well as doubts about the existence of COVID-19 itself, contributed to the hesitancy landscape in Tanzania.^7,8^

While there are high awareness levels, vaccine uptake in Tanzania remains low because of specific socio-cultural and logistical challenges, emphasising the necessity for tailored communication strategies. There is widespread awareness of COVID-19 and its associated vaccines across Tanzania. For instance, a study conducted in eight regions of Tanzania, encompassing six regions on the mainland and two in Zanzibar, revealed that 94% of participants were aware of COVID-19 vaccines; however, 75% were unable to identify any specific vaccine.^9^ However, existing studies in Tanzania have primarily focussed on vaccine hesitancy and public perceptions, leaving a gap regarding how specific communication channels influence vaccine uptake.^10,11,12^ While recent studies, such as those by Mudenda et al,^13^ and Bono et al.,^14^ have explored general attitudes towards COVID-19 vaccines in sub-Saharan Africa, few have specifically examined the effectiveness of media and community engagement strategies in promoting vaccine acceptance in Tanzania.^15^

Despite the recognised role of media and community engagement in influencing vaccine uptake, there is a lack of empirical evidence on how different communication channels specifically impact COVID-19 vaccine acceptance and uptake in Tanzania. This study aims to address this gap by examining the critical role of media and community engagement in shaping public perception and promoting COVID-19 vaccine acceptance in Tanzania, highlighting the importance of effective communication channels in the country’s vaccination efforts. It examines the specific channels used and assesses their impact on vaccine perception and acceptance. By providing empirical evidence on how these strategies impact vaccine perception and uptake, this study seeks to inform public health communication efforts. Understanding the critical roles of various communication channels is vital for improving vaccine acceptance and coverage in Tanzania. Media platforms such as radio, television and social media play a crucial role in shaping public opinion and behaviour, especially in health-related matters.^16^ The way information about COVID-19 and its vaccines is presented can significantly influence public perceptions and attitudes towards vaccination. Given that media consumption varies significantly across urban and rural areas, investigating the effectiveness of different media platforms in disseminating vaccine-related information is imperative.^15^ This study also seeks to answer the following questions: *How are the media and community engagement strategies utilised in Tanzania’s COVID-19 vaccination campaign in promoting vaccine acceptance? How have these channels influenced the vaccine uptake among the Tanzanian public?*

By addressing these questions, this study aimed to contribute to the existing knowledge on fostering vaccine uptake amid misinformation and entrenched beliefs. Tanzania offers unique challenges and opportunities for public health communication, and this study was set within a global health communication discourse.

### COVID-19 vaccinations in Tanzania: An overview

As of November 2024, Tanzania’s COVID-19 vaccination campaign has achieved significant milestones, yet challenges persist in reaching comprehensive coverage.^17^ The country commenced its vaccination efforts in July 2021, initially receiving over one million doses of the Johnson & Johnson vaccine through the COVID-19 Vaccines Global Access (COVAX) initiative with support from the United States (US). Subsequent donations included vaccines from China and additional supplies via COVAX, facilitating the inoculation of priority groups, such as healthcare workers, the elderly and individuals with underlying health conditions. By April 2023, Tanzania had vaccinated approximately 51.0% of its population, a substantial increase from the mere 2.8% coverage reported in January 2022. This progress has been attributed to intensified vaccination campaigns and enhanced public health strategies aimed at increasing vaccine uptake.^18^ Despite these advancements, Tanzania faces ongoing obstacles to achieving herd immunity. Vaccine hesitancy, fuelled by misinformation and logistical challenges in rural and remote areas, continues to impede vaccination. Efforts to integrate COVID-19 vaccination into routine healthcare services are underway with the aim of sustaining and expanding coverage.^19,20^ The government’s collaboration with international partners, including the World Health Organization (WHO) and United Nations Children’s Fund (UNICEF), remains crucial in addressing these challenges.

Tanzania’s response to COVID-19 vaccinations has been shaped by various factors including digital transformation, public communication and healthcare system challenges. The country has experienced a significant push towards adopting digital solutions (such as remote work technologies, e-commerce platforms and online education systems) during the pandemic, which has enabled businesses and individuals to cope with its impact.^11^ This digital transformation has been extended to higher learning institutions, where e-learning systems have been partially implemented, although with limited use.^21^ Interestingly, while digital technologies have been embraced in many sectors, public communication about COVID-19 in Tanzania has predominantly relied on traditional media, particularly political cartoons.^22,23,24^ These cartoons, often in Kiswahili, have been used to convey messages about the spread of the virus, preventive measures and the role of political leadership in managing the pandemic.^3^ The contrast between digital advancement and traditional communication methods highlights the complex nature of Tanzania’s response to the pandemic. Tanzania’s approach to COVID-19 vaccination appears to be influenced by a combination of digital transformation, traditional communication methods and challenges in the healthcare system. While the country has made strides in adopting digital technologies, there is a need for more comprehensive e-learning policies and strategies in higher education institutions.^21^ Moreover, the use of political cartoons as a primary means of public communication regarding the pandemic suggests the need for more diverse and inclusive information dissemination strategies.

### Media influence on vaccine acceptance

Media influence plays a significant role in public perception and acceptance of vaccines. Studies have shown that media coverage, particularly in the context of human papillomavirus (HPV) vaccination, can support vaccination by dismissing concerns about vaccines, leading to promiscuous behaviour and emphasising the vaccine’s lifesaving potential.^25^ However, media portrayals of vaccine-related controversies, such as those surrounding the HPV vaccine, can also contribute to vaccine hesitancy, with conflict-containing stories peaking during certain periods.^26^ Social media discussions reflect major events and demonstrate global perspectives with an increasingly positive sentiment and trust, potentially implying a higher acceptance of COVID-19 vaccines.^27^ Conversely, media framing strategies that manifest fear and action cues can influence vaccine acceptance, suggesting the need for targeted and effective communication campaigns.^28^ Trust in health authorities and scientists is associated with higher vaccine acceptance, whereas reliance on social media is linked to lower acceptance.^29^ The influence of the media on vaccine acceptance is complex, with both positive and negative effects. Trust in authoritative sources and constructive media coverage can boost vaccine acceptance, whereas conflict and misinformation can cause hesitancy.

### Community outreach and vaccine uptake

The effectiveness of community outreach programmes has been widely recognised in public health literature. Community-based initiatives engage local leaders, healthcare workers and volunteers in addressing vaccine concerns and providing accurate information.^30^ These programmes effectively reach underserved populations and communities with low vaccination rates.^15,31^ By tailoring messages to specific cultural contexts and addressing unique barriers (such as language differences, religious beliefs and the mistrust of medical institutions), community outreach can significantly improve vaccine uptake and contribute to broader public health goals. These programmes, which involve direct community engagement, are especially pertinent in regions with high scepticism towards external health interventions. Cyril and Smith^32^ highlighted the success of community engagement strategies that involve local leaders and healthcare workers in building trust and facilitating the acceptance of health interventions. Despite their potential benefits, community-based initiatives for vaccine promotion face significant challenges. These programmes may struggle with limited resources, inconsistent messaging across different community groups and the potential for local leaders to spread misinformation if not properly educated.^33,34^ Furthermore, relying heavily on community engagement strategies may inadvertently reinforce existing social divisions and inequalities, particularly in diverse communities with complex cultural dynamics. In Tanzania, understanding and integrating local cultural dynamics is crucial for effective health communication. Mkandawire and Muyenga^35^ point out the importance of aligning health messages with local cultural norms and values, which can significantly enhance the receptivity of these messages.

### Challenges of misinformation and trust issues in media and community outreach

Effective media and community outreach are important for addressing the challenges of misinformation and building public trust in health interventions during the COVID-19 pandemic. Misinformation spread through various media channels can erode confidence in vaccination and other health measures.^36^ While media and community outreach can be valuable tools for combating misinformation, they may also inadvertently contribute to the problem by oversimplifying complex issues or amplifying conflicting messages. Moreover, the effectiveness of these approaches can be limited by existing distrust in institutions and media outlets, potentially reinforcing scepticism rather than building confidence in health interventions.^37^ Southwell and Otero Machuca^38^ discussed strategies to combat misinformation, emphasising the need for clear, consistent and transparent communication from health authorities delivered through trusted media and community platforms. Unverified health claims such as the supposed protective properties of lemon juice against COVID-19 highlight the importance of disseminating accurate information. Trust in the sources of health information significantly affects vaccine acceptance, as illustrated by Qiao and Friedman^29^ Therefore, media and community outreach efforts must focus on enhancing the credibility of health messages and the institutions that deliver them.

Understanding the unique socio-cultural and political landscape of Tanzania is vital for the effective implementation of media and community outreach strategies. Studies by Mboera and Rumisha^39^ and Holst and Isabwe^40^ provide insights into how local contexts shape public attitudes towards health interventions. Tailoring communication strategies to address specific cultural contexts and barriers could improve public trust and counteract misinformation, ultimately contributing to broader public health goals.

## Research methods and design

### Study design and setting

This study employed a mixed-methods approach to examine the societal dynamics of COVID-19 vaccine acceptance in Tanzania. The mixed-methods approach incorporated both quantitative and qualitative data collection techniques to provide a comprehensive understanding of vaccine acceptance issues. The research design involved surveys, interviews and focus groups to capture diverse perspectives from various stakeholders in Tanzania. The study was conducted between June 2023 and August 2023.

#### Sampling frame and stratification

Participants were selected using a stratified random sampling method based on 2022 Tanzania Population and Housing Census data, which served as the sampling frame for the study. Stratification was conducted at two levels: regional and district. This study focussed on eight regions – Arusha, Mbeya, Morogoro, Mtwara, Njombe, Shinyanga, Singida and Tabora – to represent diverse geographical zones and socio-cultural contexts within Tanzania. These regions were selected considering variations in urbanisation levels, media penetration and community engagement practices. Within each region, districts were chosen to include both urban and rural areas, ensuring the representation of different residential settings. Districts were categorised based on administrative classifications provided by the National Bureau of Statistics.

#### Sampling procedure

The 2022 Tanzania Population and Housing Census provided updated population data for all regions, districts and wards. These census data served as the basis for stratifying the population into urban and rural strata. The population of each region was proportionally divided among the districts based on census data. Households were randomly selected within each district using lists obtained from the local administrative offices. Participants were chosen randomly within households using the Kish grid method to ensure an equal probability of selection and minimise bias. The urban-rural breakdown of the census data was used to allocate sample sizes proportionally within the districts. In cases where urban-rural representation differed substantially, weights were adjusted to ensure accurate representation.

#### Data collection

Quantitative data were collected through structured questionnaires administered to a representative sample of 3098 residents across eight regions of Tanzania (Table 1). The questionnaires were designed to capture demographic information, socioeconomic status, access to and trust in various media channels, experiences with community outreach initiatives and specific indicators related to the research objectives of assessing the effectiveness of media and community outreach on COVID-19 vaccination uptake.

**TABLE 1:** Geographical distribution and sampling of the mini-survey.

SN	Region	District/council	Population (2022 Census)	% of regional population	Sample size
1	Arusha	Arusha CC	1 423 932	36.3	107
		Arusha DC	1 123 073	28.2	90
		Meru DC	1 416 442	35.5	161
		Total	3 963 447	100	358
2	Mbeya	Chunya DC	1 290 478	17.1	141
		Kyela DC	1 221 490	13.0	150
		Mbeya CC	1 385 279	22.7	140
		Total	3 897 247	100	431
3	Morogoro	Ifakara TC	555 956	3.4	77
		Morogoro DC	1 286 248	17.2	141
		Morogoro MC	1 315 866	19.0	147
		Total	3 158 070	100	365
4	Mtwara	Mtwara DC	1 228 003	23.8	135
		Mtwara MC	1 108 299	11.3	142
		Nanyamba TC	1 185 174	19.3	143
		Total	3 521 476	100	420
5	Njombe	Makambako TC	993 827	8.6	103
		Makete DC	997 266	8.9	121
		Njombe TC	1 130 223	11.9	167
		Total	3 121 316	100	391
6	Shinyanga	Kishapu DC	1 272 990	21.4	124
		Shinyanga DC	1 334 417	26.2	124
		Shinyanga MC	1 161 391	12.7	121
		Total	3 768 798	100	369
7	Singida	Ikungi DC	1 272 959	23.8	126
		Singida DC	1 225 521	19.7	132
		Singida MC	1 150 379	13.1	128
		Total	3 648 859	100	386
8	Tabora	Tabora MC	1 226 999	20.3	137
		Urambo DC	1 192 781	17.3	121
		Uyui DC	1 396 623	35.6	120
		Total	3 816 403	100	378

**Grand total**		**-**	**28 895 616**	**-**	**3098**

SN, serial number; CC, City Council; DC, District Council; MC, Municipal Council; TC, Town Council.

#### Proportional allocation

The sample size for each region and district was allocated proportionally based on their population sizes relative to the total population of the eight regions as per the 2022 census data. This proportional allocation ensured that regions and districts with larger populations contributed a correspondingly larger number of participants to the sample, thus enhancing the representativeness of the findings. Table 1 illustrates how stratified random sampling was conducted, including the total population of each district, the percentage of the regional population and the allocated sample size.

#### Urban and rural selection

Within each district, the sample was further stratified to include participants from both urban and rural areas based on the urban-rural population distribution provided by the census data. Households were randomly selected from village and street registers obtained from the local administrative offices. This stratification ensured adequate representation of both urban and rural populations, allowing for a comparative analysis of media access, trust in communication channels and experiences with community outreach.

#### Data analysis

Quantitative data were analysed using Statistical Package for Social Sciences (SPSS ver27) software (SPSS Inc., Chicago, IL, US). Descriptive statistics, including measures of central tendency and variability, were calculated to provide an overview of the dataset characteristics. The findings are summarised and presented in tables and graphs, offering visual representations of the general trends and patterns within the data. This straightforward approach allowed for clear communication of the results without employing advanced statistical analyses or hypothesis testing.

### Qualitative methodology

#### Sampling and data collection

The sampling process for this qualitative study was designed to ensure the inclusion of diverse perspectives from the selected study areas. A purposive sampling strategy was employed using specific criteria to identify and recruit participants who could provide relevant insights into COVID-19 vaccine uptake. These criteria included participants’ roles in the community (e.g. healthcare workers and community leaders), vaccination status (vaccinated or unvaccinated) and representation of marginalised populations (e.g., individuals from low-income groups, women or those in rural settings). This approach was guided by the need to capture a broad range of socio-cultural contexts and life experiences. Participants were recruited from eight selected regions, with efforts made to include individuals from various socio-demographics. The study sample comprised 24 key informant interviews (KIIs) and six focus group discussion (FGDs) sessions with participants selected from two wards in three districts from each region. Inclusion criteria were established to ensure representation from key stakeholder groups, including healthcare workers, community leaders, vaccinated and unvaccinated individuals, and marginalised populations.

Participants for the FGDs included community members selected to represent diverse demographics, aiming to explore community perspectives, social norms and collective experiences related to vaccination. Key informants, comprising healthcare professionals, community leaders and local policymakers, provided in-depth insights into the local health infrastructure, policies and societal factors influencing vaccine uptake. The FGDs and KIIs were conducted in Swahili to ensure that the participants could express themselves comfortably and accurately. Audio recordings from these sessions were later transcribed and translated into English for analysis. To capture non-verbal cues and group dynamics, trained observers were present during the FGDs, taking detailed field notes to complement the collected verbal data.

#### Data analysis

Qualitative data were analysed using the NVivo software (Lumivero, Burlington, Massachusetts, US). A thematic analysis approach was employed to identify and interpret the patterns and themes within the data. The qualitative data analysis process began with the use of NVivo software, a powerful tool designed to facilitate the organisation, coding and interpretation of non-numerical data. NVivo allows researchers to efficiently manage large volumes of text-based information, audio recordings and visual materials. Thematic analysis, a widely used method in qualitative research, was applied to uncover meaningful patterns and themes within the collected data.^41^ This approach involved several iterative steps: familiarisation with the data through repeated reading, generating initial codes to identify relevant features, searching for potential themes by collating codes, reviewing and refining themes to ensure coherence, defining and naming themes to capture their essence, and producing a comprehensive report. Throughout this process, the researchers engaged in constant comparisons, moving back and forth between the raw data, codes and emerging themes to ensure a robust and grounded analysis that accurately reflected participants’ experiences and perspectives. This analysis provides an understanding of the underlying motivations, beliefs and attitudes towards the COVID-19 vaccine among different stakeholder groups.

### Ethical considerations

Ethical approval for this study was granted by the Institutional Research Review Committee of the University of Dodoma (UDOM) - (MA.84/261/76/214). Participants were provided detailed information about the study’s purpose, procedures, potential risks and benefits. Informed consent was obtained from all participants, ensuring that their involvement was voluntary and free from coercion. Confidentiality and anonymity were maintained throughout the research process, and the findings were reported accurately and without bias to reflect the true perspectives of the participants.

## Results

The findings of this study are presented in two separate sections: (1) the quantitative results derived from the structured questionnaires, followed by (2) the qualitative insights gathered from FGDs and KIIs.

### Quantitative findings

#### Awareness and perceptions

The findings indicate widespread awareness of the COVID-19 pandemic across Tanzania, although perceptions of vaccine safety and efficacy varied among regions. For example, Table 2 shows that a very high percentage of the population (99.3%) across all regions had heard of the COVID-19 pandemic. However, a minor gap in public awareness was noted, with 0.7% of the surveyed population being unaware of the pandemic. The findings also show some regional variation. For example, Arusha and Tabora had 100% awareness levels. In contrast, the Mbeya, Mtwara, Njombe, Shinyanga and Singida regions had near-universal awareness, ranging from 99.2% to 99.7%, whereas the Morogoro region had the lowest awareness rate of 97.5%, albeit still high (Table 2).

**TABLE 2:** Awareness of the COVID-19 pandemic (*N* = 3098).

Region	No		Yes		Total	
	*n*	%	*n*	%	*n*	%
Arusha	0	0.0	358	100.0	358	100.0
Mbeya	3	0.7	428	99.3	431	100.0
Morogoro	9	2.5	356	97.5	365	100.0
Mtwara	2 -	0.5	418	99.5	420	100.0
Njombe	3	0.8	388	99.2	391	100.0
Shinyanga	3	0.8	366	99.2	369	100.0
Singida	1	0.3	385	99.7	386	100.0
Tabora	0	0.0	378	100.0	378	100.0

**Total**	**21**	**0.7**	**3077**	**99.3**	**3098**	**100.0**

#### Vaccination status and factors affecting vaccination decisions

Table 3 provides data on COVID-19 vaccination rates across the eight regions in Tanzania. These data suggest that there are significant regional differences in vaccine uptake. For example, Mtwara stands out with an equal split of 50% vaccinated and 50% unvaccinated residents, whereas Morogoro showed the highest percentage of unvaccinated individuals at 77.5%. Overall, combining data from all regions, 37.2% of the total surveyed population had been vaccinated against COVID-19 and 62.8% did not.

**TABLE 3:** Vaccination against the COVID-19 (*N* = 3098).

Region	Have you heard about the COVID-19 pandemic?				Total	
	No		Yes			
	*n*	%	*n*	%	*n*	%
Arusha	224	62.6	134	37.4	358	100.0
Mbeya	318	73.8	113	26.2	431	100.0
Morogoro	283	77.5	82	22.5	365	100.0
Mtwara	210	50.0	210	50.0	420	100.0
Njombe	248	63.4	143	36.6	391	100.0
Shinyanga	230	62.3	139	37.7	369	100.0
Singida	194	50.3	192	49.7	386	100.0
Tabora	239	63.2	139	36.8	378	100.0

**Total**	**1946**	**62.8**	**1152**	**37.2**	**3098**	**100.0**

#### Healthcare providers’ engagement in COVID-19 vaccination benefits

Healthcare providers significantly informed the public about COVID-19 vaccination benefits, with 77.9% of the respondents acknowledging receiving information. However, 22.1% of the participants did not receive this information. The data in Table 4 show disparities in education levels across regions, with Singida having the highest proportion of informed respondents (91.2%) and Morogoro the lowest (66.3%).

**TABLE 4:** Healthcare providers’ engagement on the COVID-19 vaccination (*N* = 3098).

Region	No		Yes		Total	
	*n*	%	*n*	%	*n*	%
Arusha	61	17.0	297	83.0	358	100.0
Mbeya	124	28.8	307	71.2	431	100.0
Morogoro	123	33.7	242	66.3	365	100.0
Mtwara	108	25.7	312	74.3	420	100.0
Njombe	90	23.0	301	77.0	391	100.0
Shinyanga	80	21.7	289	78.3	369	100.0
Singida	34	8.8	352	91.2	386	100.0
Tabora	64	16.9	314	83.1	378	100.0

**Total**	**684**	**22.1**	**2414**	**77.9**	**3098**	**100.0**

#### Communication channels and community engagement

Community leaders significantly influenced public perceptions of COVID-19 and vaccinations, informing the public about the importance of vaccination and recognising the threat. Table 5 shows that out of the 3098 respondents who were not vaccinated against COVID-19, 52.1% (*n* = 1615) reported that their decision was influenced by their political, religious or community leader. Meanwhile, 37.2% (*n* = 1152) stated that their decisions were not influenced by such leaders, and 10.7% (*n* = 331) were unsure. Regionally, Morogoro had the highest percentage of respondents who felt influenced by leaders (65.5%, *n* = 239), while Mtwara had the lowest percentage of respondents 37.6% (*n* = 158). Mtwara also had the highest proportion of respondents who said ‘no’ to leader influence, at 50.0% (*n* = 210). The percentage of respondents who were ‘not sure’ varied across regions, with Mbeya having the highest at 21.8% (*n* = 94) and Arusha the lowest at 2.5% (*n* = 9). These figures indicate regional variations in the perceived influence of leaders on individuals’ decisions not to get vaccinated.

**TABLE 5:** Influence of political, religious or community leaders on unvaccinated individuals’ decisions regarding the COVID-19 vaccination (*N* = 3098).

Region	No		Since you were not vaccinated against COVID-19, was your decision influenced by your political, religious or community leader?				Total	
			Yes		Not sure			
	*n*	%	*n*	%	*n*	%	*n*	%
Arusha	134	37.4	215	60.1	9	2.5	358	100.0
Mbeya	113	26.2	224	52.0	94	21.8	431	100.0
Morogoro	82	22.5	239	65.5	44	12.1	365	100.0
Mtwara	210	50.0	158	37.6	52	12.4	420	100.0
Njombe	143	36.6	177	45.3	71	18.2	391	100.0
Shinyanga	139	37.7	206	55.8	24	6.5	369	100.0
Singida	192	49.7	183	47.4	11	2.8	386	100.0
Tabora	139	36.8	213	56.3	26	6.9	378	100.0

**Total**	**1152**	**37.2**	**1615**	**52.1**	**331**	**10.7**	**3098**	**100.0**

Table 6 presents the frequencies and percentages of respondents’ most trusted sources of information regarding COVID-19 vaccination. According to the data, radio is the most trusted source, mentioned by 70.6% of the respondents (*n* = 358). Television was the second-most cited source, accounting for 58.0% (*n* = 294). Health workers were trusted by 41.8% of the respondents (*n* = 212), followed by religious leaders at 34.7% (*n* = 176). Social media is a trusted source for 23.7% of the respondents (*n* = 120). Other sources include family and friends (19.3%, *n* = 98), community leaders (16.6%, *n* = 84) and political leaders (11.8%, *n* = 60). Lesser-mentioned sources are health facilities (9.5%, *n* = 48), newspapers and magazines (7.1%, *n* = 6), village meetings (6.3%, *n* = 32), public announcements (5.5%, *n* = 28), posters and leaflets (3.9%, *n* = 20), SMS messages (2.4%, *n* = 12 mentions) and don’t know (1.6%, *n* = 8).

**TABLE 6:** Trusted sources of information about the COVID-19 vaccination (*N* = 507).

Trusted source	Frequency (*n*)	%
Radio	358	70.6
Television	294	58.0
Health workers	212	41.8
Religious leaders	176	34.7
Social media	120	23.7
Family and friends	98	19.3
Community leaders	84	16.6
Political leaders	60	11.8
Health facilities	48	9.5
Newspapers/magazines	36	7.1
Village meetings	32	6.3
Public announcements	28	5.5
Posters/leaflets	20	3.9
SMS messages	12	2.4
Don’t know	8	1.6
Other	6	1.2

**Total**	**507†**	**100.0**

† , The total number represents the cumulative count of all the sources mentioned. As respondents could list up to three sources each, the total could not be the same as the sample size for the survey.

### Qualitative findings

#### Awareness and perceptions of COVID-19 and vaccination

Awareness of COVID-19 was widespread, but perceptions about the disease and its vaccine varied significantly across regions and were often influenced by local myths and inconsistent communication. In Mtwara, for instance, one participant reflected the common scepticism, stating:

‘On the streets, we were told that if you get vaccinated, you might turn into a dog … but I trust our government wouldn’t bring anything harmful to its citizens.’ (KII9, Ward Executive Officer, Male, 43 years, Mtwara)

This scepticism is not limited to Mtwara, as similar misconceptions have been reported in other regions. Despite these challenges, some participants expressed trust in the government-endorsed health initiatives. The varying levels of awareness and perceptions underscored the need for targeted and culturally sensitive communication strategies to address local concerns and promote accurate information about COVID-19 and vaccination.

However, fear of side effects and mistrust of the vaccine’s origins remain prevalent. In Njombe, a participant shared their hesitation, noting:

‘I can’t vaccinate because the people out there don’t wish us well … Why can’t our Tanzanian experts make the vaccines for us?’ (KII6, Small Farmer, Female, 26 years, Njombe)

Such sentiments reflect broader uncertainties stemming from misinformation and cultural beliefs, which are compounded by social media rumours and inconsistent messaging from political leaders.

#### Government trust and information dissemination

Trust in government communication plays a critical role in influencing vaccination decisions, although it is not universal. In Tabora, a respondent highlighted how influential government and religious leaders were in promoting vaccination, saying:

‘The reason why I got vaccinated, I saw the president getting vaccinated, and I said if the president is getting the vaccine, why not me?’ (FGD13, Businessman, Male, 50 years, Tabora)

Similarly, in Shinyanga, the effectiveness of information dissemination through vehicles with audible sound and music was emphasised by many respondents, who noted that it reached even remote areas: ‘I have observed that vehicles with loudspeakers and music successfully carry our messages to even the most remote parts of Shinyanga’ (KII7, Hamlet Chairperson, Male, 54 years, Shinyanga).

On the other hand, inconsistencies in government statements created confusion in regions such as Singida. One participant remarked:

‘The decision not to vaccinate arose because the fifth government administration said one thing, and the sixth said something different.’ (KII5, Village Executive Officer, Male, 55 years, Singida)

This eroded confidence and emphasised the importance of clear and consistent messaging in building public trust. These inconsistencies in government statements created confusion and highlighted the need for a unified approach to public health communication. The impact of such mixed messages extends beyond immediate confusion, potentially undermining long-term trust in health initiatives and vaccination programmes.

#### Dissecting regional variations in vaccine uptake and decision-making

Regional disparities in vaccine uptake were pronounced and shaped by access to healthcare, cultural beliefs and economic factors. In Morogoro, logistical challenges were a major barrier, with one resident explaining:

‘We decided to go, but due to the long queues, we gave up.’ (KII2, Carpenter, Male, 30 years, Morogoro)

Further analysis revealed that rural areas face additional hurdles, including limited transportation options and inadequate information dissemination regarding vaccine availability. Social media has played a significant role in shaping perceptions, with misinformation spreading rapidly in some communities and influencing decision-making processes. Local leaders and healthcare workers emerged as important influencers, and their endorsement or scepticism had a substantial impact on community attitudes towards vaccination. Rural communities faced even greater hurdles, such as long travel distances and inadequate vaccine supply, which discouraged many from seeking vaccination, as reported in the Arusha and Singida regions.

The cultural attitudes varied significantly. In Mtwara, traditional remedies were still viewed as sufficient, with one participant claiming, ‘We used our natural medicines even before sanitizer’ (FGD4, Village Chairperson, Female, 38 years, Njombe). The influence of social media extended beyond shaping perceptions, as it became a platform for organising grassroots initiatives to promote vaccination awareness and combat misinformation. Healthcare workers and local leaders have leveraged these digital platforms to reach wider audiences and provide accurate information about vaccine safety and efficacy. Moreover, some health workers in Arusha and Mbeya have developed innovative solutions to address the challenges faced by rural areas, such as sending mobile text messages and establishing vaccination clinics to improve access for those living in remote locations. Conversely, urban regions such as Shinyanga saw higher vaccination rates, bolstered by the presence of influential community leaders and better healthcare access: ‘I have seen firsthand how respected community leaders and improved healthcare access in urban Shinyanga are clearly elevating vaccination rates’ (KII14, Small Farmer, Female, 48 years, Shinyanga).

#### Empowering healthcare providers to bridge vaccine information gaps

Healthcare providers have emerged as pivotal but polarising figures in the vaccination process. In Tabora, some health workers were cited as discouraging vaccination, with one respondent recounting, ‘Health workers told us that these vaccines are not safe’ (FGD3, Shopkeeper, Male, 49 years, Tabora). This undermined public confidence and created confusion regarding the vaccine efficacy. This inconsistency in messaging from healthcare professionals significantly affected the vaccine uptake rates in the region. Local community leaders stepped in to bridge the information gap, organise door-to-door campaigns to address concerns and provide accurate vaccine information. Despite these efforts, the initial damage to public trust proved challenging to overcome, highlighting the critical need for unified, science-based communication from all healthcare providers.

However, proactive health workers who were actively engaged in communities showed a positive difference. A participant in Shinyanga noted, ‘Healthcare workers came to the market and encouraged us’ (KII18, Book Seller, Male, 22 years, Shinyanga). These community-based initiatives not only helped disseminate accurate information but also created a sense of trust and personal connection with the vaccination campaign. The presence of healthcare workers in familiar settings, such as markets and neighbourhoods, made the vaccine more accessible and relatable to the local population. This grassroots approach demonstrates the importance of tailoring communication strategies to specific community needs and cultural contexts, potentially offering valuable lessons for future public health initiatives.

#### Enhancing vaccine uptake through community leader engagement

Community leaders, particularly religious and traditional figures, were instrumental in influencing perceptions of vaccines. In Tabora, a respondent shared, ‘Through our President and the Mufti … I believed as a follower I should vaccinate’ (KII5, Village Executive Officer, Female, 33 years, Tabora). This underscores the power of leadership in swaying public attitudes towards vaccination. This influence extended beyond religious leaders to include local government officials and respected community elders. These influential figures often acted as role models by publicly receiving vaccinations themselves, thereby encouraging others to follow suit. Their endorsement of vaccination campaigns helped dispel misconceptions and build trust within the community, leading to increased vaccine acceptance and uptake.

However, in some regions, the absence of an active leader involvement creates a vacuum. One participant remarked, ‘There is a lack of cooperation between our ward leaders and us citizens’ (FGD2, Banana Seller, Male, 26 years, Morogoro). Participants emphasised the need for leaders to work closely with healthcare teams to dispel myths and encourage vaccination, particularly in rural areas where formal communication channels are less effective. This absence of active leadership involvement in some regions, such as the Mbeya and Mtwara regions, highlights the critical role that local leaders play in successful vaccination campaigns. It was reported that FGDs and KIIs without their support, misinformation and hesitancy spread more easily, potentially undermining public health efforts.

## Discussion

### Awareness and perceptions of COVID-19 and vaccination

The high level of awareness about COVID-19 across Tanzania is commendable, as shown in Table 1, with 99.3% of the population being informed about the pandemic. This indicates the effective dissemination of information through various channels, including television, Instagram and traditional media such as radio. However, regional variations in the perceptions of vaccine safety and efficacy highlight a critical gap. Regions such as Arusha and Tabora achieved a 100% awareness level, while Morogoro lagged at 97.5%. These discrepancies could be attributed to differences in communication strategies or media access across regions. These regional disparities in vaccine awareness and perception underscore the need for tailored communication strategies to address local concerns and cultural contexts. Policymakers and health officials could benefit from analysing the successful approaches employed in high-awareness regions such as Arusha and Tabora to inform and improve outreach efforts in areas with lower awareness levels. Moreover, investigating specific barriers to information dissemination in regions such as Morogoro could provide valuable insights for developing more effective and targeted public health communication campaigns. This finding aligns with the communication model proposed by Nan and Iles,^42^ which emphasises the need for tailored public health messaging to address diverse regional contexts.

### Government trust and information dissemination

The study revealed a high level of trust in the government as a reliable source of COVID-19 information, with traditional media outlets such as radio and television being the most trusted sources. This finding resonates with the work of Majid and Wasim,^43^ who underscored the role of government and media in fostering public trust during health crises. However, the regional variances in this trust signal a need for more nuanced, culturally sensitive and region-specific communication strategies to address the diverse information needs and preferences of people. The Tanzanian population has varied access to information, with disparities based on geographical location, educational level and socioeconomic status. Tailoring communication strategies that are inclusive and ensuring that remote and underprivileged communities have access to credible information are pivotal.^44^

Misinformation remains a pervasive challenge for enhancing vaccine uptake. Misconstrued narratives, sometimes fuelled by political undertones or misinformation campaigns on social media platforms, have fostered scepticism towards the vaccine.^45^ For example, a recent study in the US found that mixed or changing messages from health authorities during the pandemic exacerbated doubts and confusion, further eroding trust.^5^ Addressing this demand is a concerted effort to foster media literacy and promote evidence-based discourse through all the communication channels. Media literacy programmes have emerged as vital instruments for curbing misinformation and fostering a society capable of critical thinking and discerning credible information sources. Engaging the educational sector and community-based organisations in Tanzania to promote media literacy could pave the way for a more informed and resilient society.^46^ Thus, this model demonstrates the importance of clear and accurate communication about vaccines, even when vaccination is mandatory and the resulting coverage is high, to reduce the spread of inaccurate information that can foster vaccine hesitancy and hinder the uptake of future vaccines.^47^

### Dissecting regional variations in vaccine uptake and decision-making

The regional disparities in COVID-19 vaccine uptake, as illustrated in Table 2, suggest a complex interplay between factors influencing vaccination decisions. Regions such as Mtwara and Singida, which showed higher vaccination rates (around 50%), potentially indicate more effective communication and community engagement strategies. In contrast, Morogoro and Mbeya exhibited lower vaccination rates (22.5% and 26.2%, respectively), indicating potential challenges for information dissemination. Understanding local contexts and community dynamics is pivotal in health communication as it ensures the relevance and effectiveness of public health interventions. Holst and Isabwe^40^ emphasise the significance of tailoring health messages to local languages and cultural settings, enhancing their accessibility and impact in rural communities. In addition, these scholars highlight that acknowledging and addressing the unique challenges and historical experiences of specific communities, such as distrust in medical systems, is crucial for successful health communication. In Tanzania, cultural beliefs and traditional narratives often play a considerable role in shaping individuals’ health behaviours.^48^ Respecting narratives and understanding culturally sensitive communication strategies are crucial. Collaborating with local influencers and leaders can help overcome barriers and resonate with population values.

### Empowering healthcare providers to bridge vaccine information gaps

Healthcare providers play a crucial role in educating the public about vaccination, as evidenced by the majority of respondents acknowledging that they have received information from these providers. However, the regional disparity in the levels of education or information provided, with Singida having the highest informed rate (91.2%) and Morogoro the lowest (66.3%), suggests variability in the healthcare infrastructure and community engagement strategies. This finding supports the arguments by Nkya et al.^49^, who emphasised the critical role of healthcare providers in health education and the need for adequate training and resources. Healthcare providers play a critical role in addressing vaccine hesitancy by being trusted sources of information, thus having the opportunity to influence individuals’ decisions regarding vaccination.^43^ Their approach, including using effective communication strategies like motivational interviewing, can support autonomy, reduce defensiveness and help patients make informed decisions about their health.

### Media, political leadership and community engagement in enhancing COVID-19 vaccination uptake in Tanzania

Media, political leadership and community engagement have played critical roles in shaping public perceptions and attitudes towards COVID-19 vaccination in Tanzania.^50^ The country’s experience highlights both challenges and opportunities in leveraging these channels for an effective vaccine rollout. Political factors significantly influenced public attitudes towards COVID-19 vaccines. The change in executive leadership from a denialist president to one who accepted vaccines and promoted transparency was a key facilitator of mass vaccination efforts in Tanzania.^51^ This political shift helped to create an environment that was more conducive to vaccine acceptance. However, divergent communications from consecutive presidents led to community confusion, highlighting the importance of consistent messaging from political leaders.^7,18^

The study shows that community leaders, particularly religious and traditional figures, were instrumental in influencing perceptions of vaccines. The influence extended beyond religious leaders to include local government officials and respected community elders. These influential figures often acted as role models by publicly receiving vaccinations themselves, encouraging others to follow suit. Their endorsement of vaccination campaigns helped dispel misconceptions and build trust within the community, leading to increased vaccine acceptance and uptake. However, in some regions, the absence of active leader involvement creates a vacuum. Participants emphasised the need for leaders to work closely with healthcare teams to dispel myths and encourage vaccination, particularly in rural areas where formal communication channels are less effective. The role of media, particularly social media, in shaping vaccine attitudes cannot be overstated.^52,53^ Research has shown that frequent social media exposure and interpersonal discussions are positively associated with vaccination intention in other contexts.^54,55^ This suggests that leveraging social media platforms could be an effective strategy to promote vaccine uptake in Tanzania.

The study found that radio (70.6%) and television (58.0%) were the most trusted sources of information about COVID-19 vaccination, highlighting the enduring role of traditional mass media in health communication, particularly in developing contexts such as Tanzania. This is consistent with evidence from Wakefield,^56^ who emphasised that mass media campaigns are effective tools for health promotion because of their ability to reach large audiences with repetitive messaging, which is essential for influencing public attitudes and behaviours. Similarly, Vaughan and Tinker^57^ noted that, in low-resource settings, radio and television serve as critical communication channels, particularly for rural and semi-urban populations, owing to their wide accessibility and affordability. These findings underscore the value of integrating mass media into vaccination campaigns, especially in regions with limited Internet connectivity, where digital platforms may not have a significant reach.

The trust placed on health workers (41.8%) and religious leaders (34.7%) further reflects the importance of interpersonal and community-based communication channels in promoting vaccination. Health workers are often perceived as credible sources of health information because of their professional expertise and proximity to the community, as supported by the WHO (2018), which underscores their role in building trust in health interventions. Similarly, religious leaders are pivotal in contexts where religion strongly influences community norms and values. A study by Jalloh et al. (2018) during the Ebola outbreak in Sierra Leone demonstrated that engaging religious leaders significantly increased acceptance of health interventions. These findings suggest that combining mass media with interpersonal channels can address both awareness and trust barriers, creating a more comprehensive strategy for promoting vaccine uptake.

Challenges such as misinformation about vaccines containing satanic elements and a lack of trust in coronavirus vaccines were identified, indicating the need for targeted efforts to combat misinformation. The study also revealed that having community sources of accurate information is critical to mass vaccination. This underscores the importance of engaging local community leaders and trusted information sources in vaccine communication. Community engagement strategies in Tanzania have several challenges.^31,58^ Patriarchal gender dynamics and low-risk perceptions have also been identified as barriers to vaccine uptake.^59^ These findings highlight the need for innovative community engagement strategies and region-specific interventions to improve comprehensive knowledge and address community perceptions and attitudes towards COVID-19 vaccines.^60,61^ The importance of tailoring communication channels to specific communities is further emphasised by research showing that geographical region, residence area, COVID-19 disease risk perception and good knowledge of COVID-19 vaccines are significantly associated with vaccine confidence.^22,58^ This suggests that a one-size-fits-all approach to media and community engagement may be ineffective. Tailoring messages to address specific community needs and involving local leaders in communication efforts may enhance acceptance of the vaccine.

### Limitations

This study has some limitations that could affect the validity and generalisability of the findings, including sample representativeness, language barriers and potential response biases. The sample may not fully capture Tanzania’s diverse socio-cultural landscape, which reflects the entire population. Language barriers and translation issues could have resulted in the loss or alteration of original meanings. Stratified sampling techniques were used to ensure sample representativeness, address language barriers and minimise translation issues. Professional translators were engaged in preserving their original meaning. Confidentiality and anonymity were ensured to reduce potential response biases.

## Conclusion

This study provides critical insights into the interplay between media and community engagement strategies in influencing COVID-19 vaccine perceptions and uptake in Tanzania. The findings highlight the importance of tailoring communication approaches to address the specific cultural and contextual factors that shape vaccine attitudes. Future research should explore the long-term impact of these strategies on vaccine acceptance and public health outcomes in Tanzania. Moreover, the lessons learned from this study may be applicable to other low- and middle-income countries that face similar challenges in vaccine rollout and public health communication. While the awareness of COVID-19 is nearly universal, regional disparities in vaccine acceptance highlight the limitations of broad, generalised communication strategies. Factors such as cultural beliefs, access to healthcare and credibility of information sources significantly influence public behaviour.

These findings emphasise the necessity for targeted region-specific outreach strategies. Effective communication should prioritise integrating mass media campaigns with interpersonal outreach between healthcare providers and community leaders. Addressing misinformation and fostering trust in the government and health authorities are pivotal for overcoming vaccine hesitancy. Future efforts should include media literacy programmes and proactive involvement of local influencers to enhance public understanding and confidence in health initiatives. This tailored approach has the potential to improve vaccine uptake and inform broader public health strategies, thereby ensuring a more inclusive and effective response to health challenges.
